# Supplementary material for: Novel Genetic Loci from Triticum timopheevii Associated with Gluten Content Revealed by GWAS in Wheat Breeding Lines
Source: Int J Mol Sci. 2023 Aug 27;24(17):13304. doi: 10.3390/ijms241713304 (PMC10487702; doi:10.3390/ijms241713304)
Supplement: Supplementary file 1 [file ijms-24-13304-s001.zip › Figure S1.pdf]

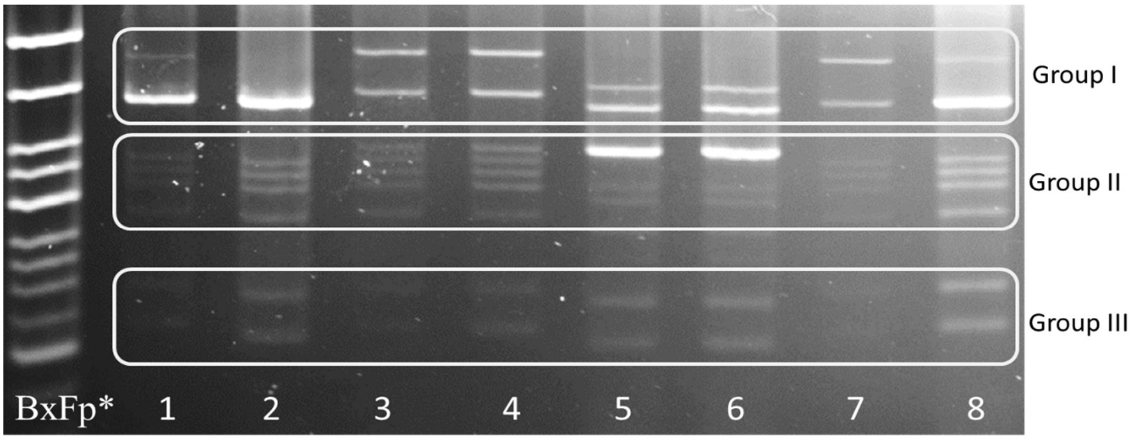

Figure S1. The electrophoregram of PCR products obtained using the BxFp* marker designed to distinguish Bx6, Bx7 and Bx7* subunits. Lines: 1 – cv. Chinese spring (Bx7); 2 – IL-676 (Bx7*); 3) IL-190/5-3 (Bx-Td); 4 – IL-190/6-1 (Bx-Td); 5 – *T. timopheevii* (Bx-Tt); 6 – *T. kiharae* (Bx-Tt); 7- *T. dicoccum* (Bx7); 8 – *T. dicoccoides* (Bx7). DNA ladder 1000 bp. PCR conditions are shown in the Table S2.
