# Supplementary material for: Novel Genetic Loci from Triticum timopheevii Associated with Gluten Content Revealed by GWAS in Wheat Breeding Lines
Source: Int J Mol Sci. 2023 Aug 27;24(17):13304. doi: 10.3390/ijms241713304 (PMC10487702; doi:10.3390/ijms241713304)
Supplement: Supplementary file 1 [file ijms-24-13304-s001.zip › Figure S2.pdf]

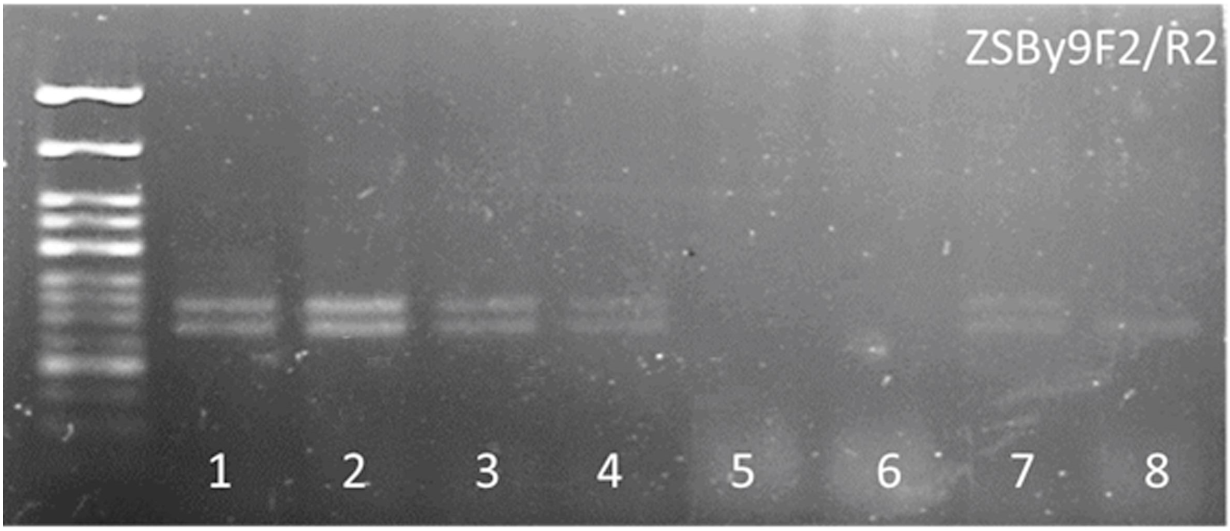

a

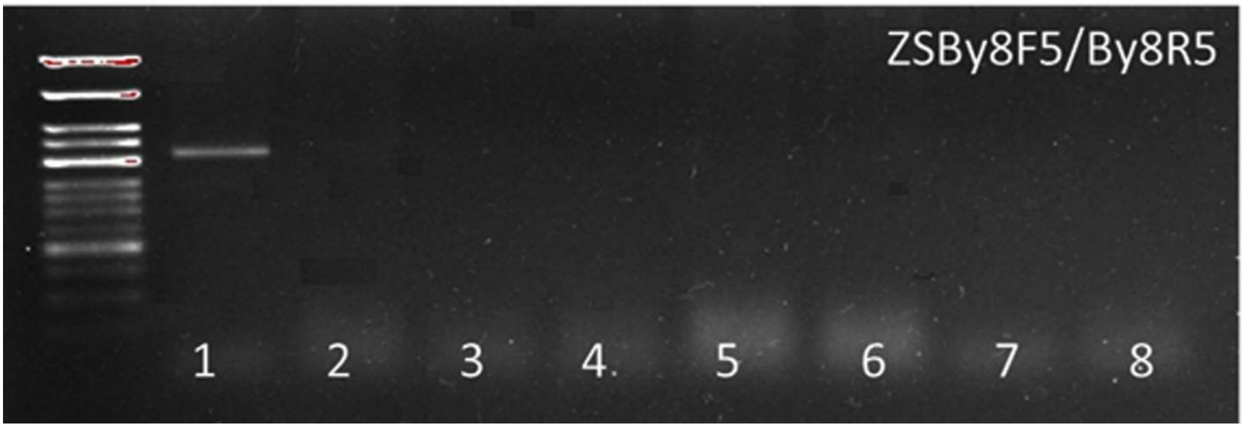

b

Figure S2. The electrophoregram of PCR products obtained with markers ZSBy9F2/R2 (a) and ZSBY8F5/By8R5 (b) developed for identification of By subunits. Lines: 1 – cv. Chinese spring (By8); 2 – IL 676 (By8*/18/15); 3) IL 190/5-3 (By8*/18/15); 4 – IL 190/6-1 (By8*/18/15); 5 – *T. timopheevii* (non-*By*); 6 – *T. kiharae* (non-*By*); 7- *T. dicoccum* (*By8*/18/15*); 8 – *T. dicoccoides* (*By18/26)*. DNA ladder 1000 bp. PCR conditions are shown in the Table S2.
