# Supplementary material for: Novel Genetic Loci from Triticum timopheevii Associated with Gluten Content Revealed by GWAS in Wheat Breeding Lines
Source: Int J Mol Sci. 2023 Aug 27;24(17):13304. doi: 10.3390/ijms241713304 (PMC10487702; doi:10.3390/ijms241713304)
Supplement: Supplementary file 1 [file ijms-24-13304-s001.zip › Figure S4.pdf]

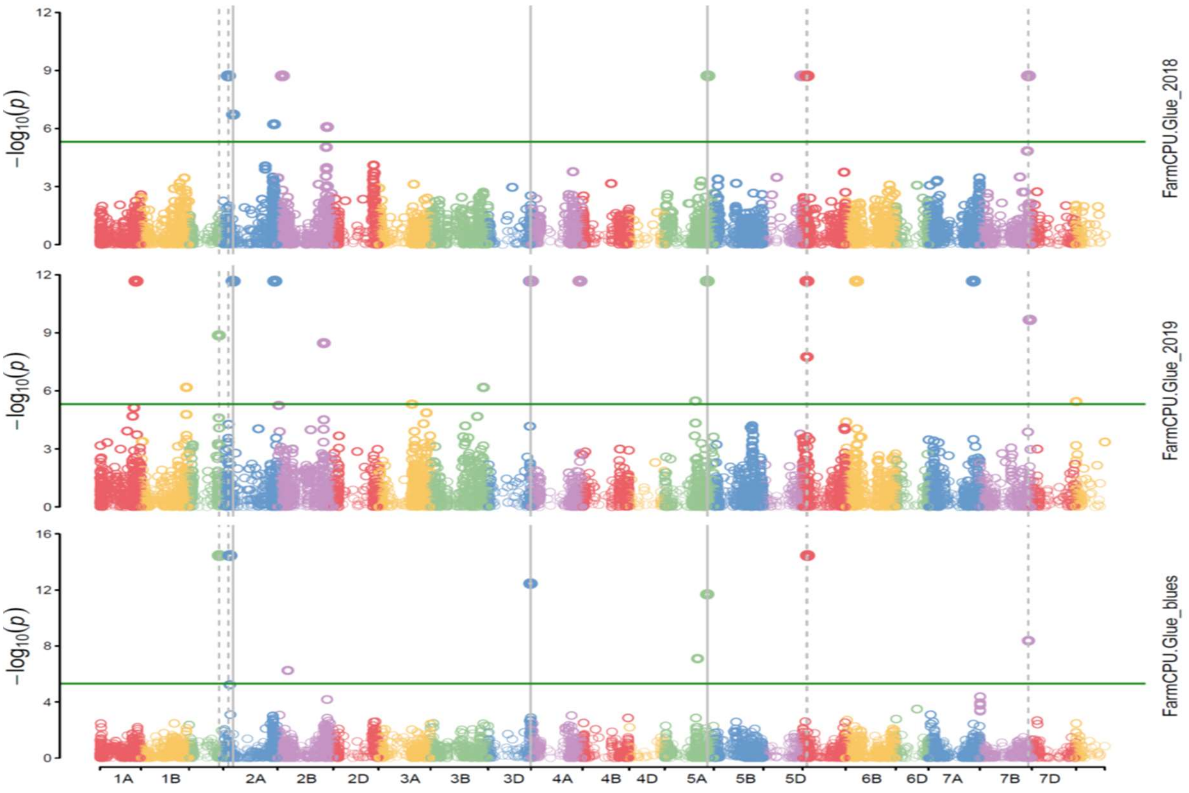

Figure S4. Manhattan plots illustrating the distribution of MTAs across chromosomes, according to the results of GC evaluation in 2018, 2019 and BLUEs.
