# Supplementary material for: Novel Genetic Loci from Triticum timopheevii Associated with Gluten Content Revealed by GWAS in Wheat Breeding Lines
Source: Int J Mol Sci. 2023 Aug 27;24(17):13304. doi: 10.3390/ijms241713304 (PMC10487702; doi:10.3390/ijms241713304)
Supplement: Supplementary file 1 [file ijms-24-13304-s001.zip › Figure S5.pdf]

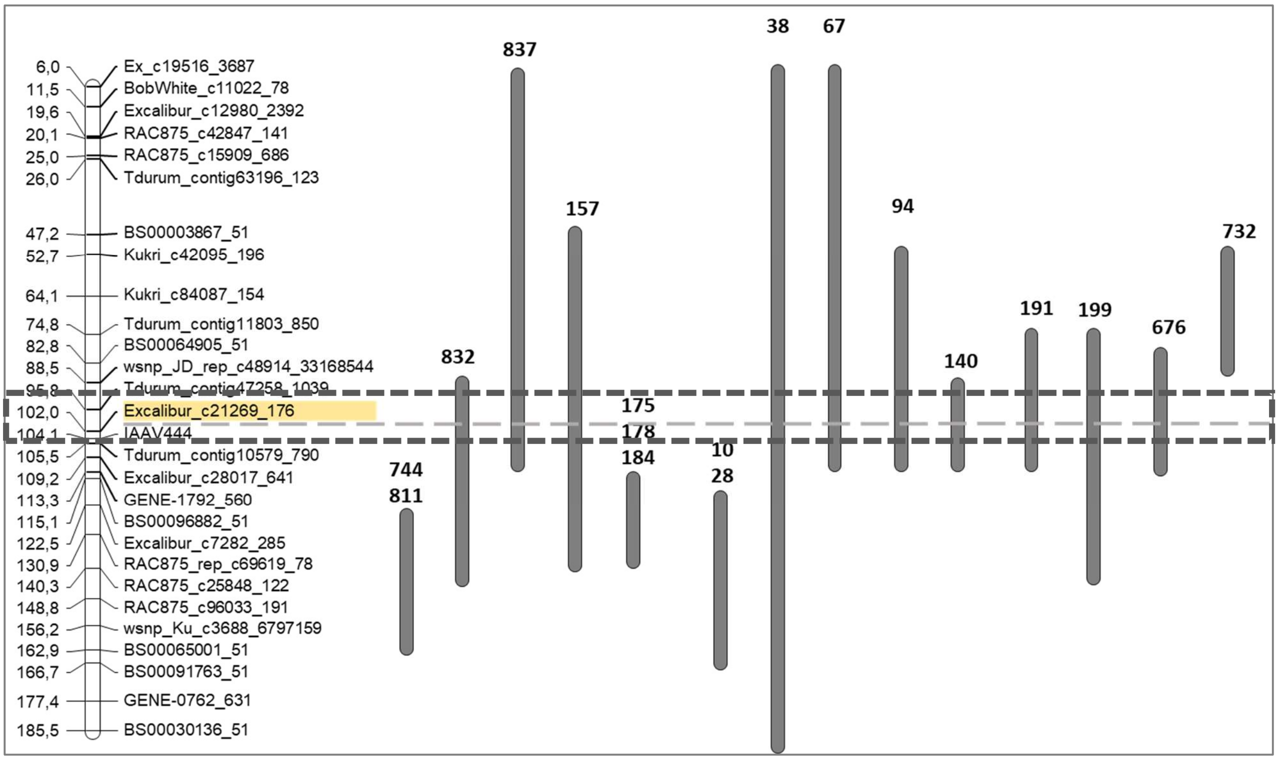

Figure S5. Schematic illustration of the position of introgressed fragments in *T. aestivum/T. timopheevi* lines. The order of the markers corresponds to the chromosome 2A consensus map for SNP markers [51]. To the left of the chromosome are the distances between markers in centiMorgans. The color indicates the most significant SNP marker. The numbers above the introgression fragments indicate the number of the IL (Table S1). Dashed line denotes a chromosome fragment common to lines with a haplotype TT/AA/GG.
